# Metronidazole Topically Immobilized Electrospun Nanofibrous Scaffold: Novel Secondary Intention Wound Healing Accelerator

**DOI:** 10.3390/polym14030454

**Published:** 2022-01-23

**Authors:** Ahmed A. El-Shanshory, Mona M. Agwa, Ahmed I. Abd-Elhamid, Hesham M. A. Soliman, Xiumei Mo, El-Refaie Kenawy

**Affiliations:** 1 Composites and Nanostructured Materials Research Department, Advanced Technology and New Materials Research Institute (ATNMRI), City of Scientific Research and Technological Applications (SRTA-City), New Borg Al-Arab, Alexandria 21934, Egypt; ahm_ch_ibr@yahoo.com (A.I.A.-E.); h.soliman@srtacity.sci.eg (H.M.A.S.); 2 Department of Chemistry of Natural and Microbial Products, National Research Center, Dokki, Giza 12622, Egypt; magwa79@gmail.com; 3 Key Laboratory of Science and Technology of Eco-Textile, Ministry of Education, College of Chemistry, Chemical Engineering and Biotechnology, Donghua University, Shanghai 201620, China; xmm@dhu.edu.cn; 4 Polymer Research Group, Chemistry Department, Faculty of Science, Tanta University, Tanta 31527, Egypt

**Keywords:** electrospinning, nanofibrous scaffold, biocompatibility, metronidazole, secondary intention wound healing, antibacterial activity

## Abstract

The process of secondary intention wound healing includes long repair and healing time. Electrospun nanofibrous scaffolds have shown potential for wound dressing. Biopolymers have gained much attention due to their remarkable characteristics such as biodegradability, biocompatibility, non-immunogenicity and nontoxicity. This study anticipated to develop a new composite metronidazole (MTZ) immobilized nanofibrous scaffold based on poly (3-hydroxy butyrate) (PHB) and Gelatin (Gel) to be utilized as a novel secondary intention wound healing accelerator. Herein, PHB and Gel were mixed together at different weight ratios to prepare polymer solutions with final concentration of (7%), loaded with two different concentrations 5% (Z_1_) and 10% (Z_2_) of MTZ. Nanofibrous scaffolds were obtained by manipulating electrospinning technique. The properties of MTZ immobilized PHB/Gel nanofibrous scaffold were evaluated (SEM, FTIR, TGA, water uptake, contact angle, porosity, mechanical properties and antibacterial activity). Additionally, in vitro cytocompatibility of the obtained nanofibrous scaffolds were assessed by using the cell counting kit-8 (CCK-8 assay). Moreover, in vivo wound healing experiments revealed that the prepared nanofibrous scaffold highly augmented the transforming growth factor (TGF-β) signaling pathway, moderately suppressed the pro-inflammatory cytokine (IL-6). These results indicate that MTZ immobilized PHB/Gel nanofibrous scaffold significantly boost accelerating secondary intention wound healing.

## 1. Introduction

Human skin is the organ most susceptible to environmental factors, and is considered to be an efficient outer most defense line  protecting the body against microorganism’s infection [1,2].

Generally, wound healing process passes through a series of physiological proceedings which include five major segments, coagulation, granulation tissue formation, re-epithelialization and extracellular matrix (ECM) remodeling [3]. Recently, multidisciplinary surgical strategies, including autograft, allograft and xenograft are employed for wound healing. In spite of their success, their application has been limited due to some drawbacks such as antigenicity, bacterial infection that hinder and delay healing process [4,5]. 

Therefore, the development of an alternative approach, wound dressings loaded with antimicrobials that protect against bacteria, accelerate wound healing and accomplish tissue regeneration has received great attention from researchers. These modern wound dressings have controlled release property of loaded antimicrobials to control bacterial growth and maintain safe concentration on tissue for accomplishing wound healing process [5,6,7]. Recently, a wide range of antibiotics and materials with antimicrobial activity have been extensively used serving as an effective wound dressing [5,6,8,9,10,11,12,13]. 

Ideal 3D tissue engineered scaffold serving as an alternative for the native tissue should provide a suitable microenvironment that satirists extracellular matrix (ECM) for a proper tissue repair [14,15]. For instance, it should provide some characteristics facilitating wound healing process such as low toxicity, high porosity, biocompatibility and suitable absorbability to remove extra exudates, assure water vapor permeability to enhance proliferation and cells spreading on the wound site and retains protection from microorganisms invasion [7,16,17,18,19,20]. As a consequence of their biocompatibility, biodegradability, hydrophilicity, low cytotoxicity, low immunogenicity and low cost, a wide range of both naturally derived and synthetic electrospun biopolymers have been prosperous in wound healing, biomedical and dental applications [6,7,21,22,23,24,25,26,27].

Among several biomaterials, polyhydroxybutyrate (PHB), a natural hydrophobic polymeric biomaterial which have been extensively used in multiple biomedical applications as tissue engineering, drug delivery [28,29,30,31,32,33], controlled release system [34] and wound dressing [35] due to its unique properties such as biodegradability, no toxicity, biocompatibility and high mechanical strength. Additionally, PHB has also been found to support human keratinocyte cell line growth and proliferation which proves its probability to be an alternative for dermal skin [36]. However, other properties of PHB such as brittleness, high crystallinity, hydrophobicity as well as high cost restricts its application as a wound dressing material [37]. For that reason, blending of PHB with other polymers (co-electrospinning) is an approach to overcome its limitations [6,33,38,39,40].

Gelatin, a natural high polymer material derived from hydrolyzed or heat denatured collagen in connective tissues or animals epidermal tissue [41]. It is similar to collagen in extra cellular matrix (ECM) of human body in composition and structure [42,43,44,45]. Due to its biocompatibility, hydrophilicity, plasticity, biodegradability, non-antigenicity, hemostatic properties and low price; gelatin has been widespread used in tissue regeneration and wound dressing applications [6,46,47,48,49,50,51,52,53,54].

Electrospun nanofibers serving in multiple prevailing applications such as, medical and biomedical applications, biosensors, sensors fabrication, water purification and environmental protection [55,56,57,58,59,60]. Additionally, electrospun nanofibers provides multiple advantages as, structure simulating skin ECM, high porosity and large surface to volume ratio which allow encapsulation of various drugs with controlled drug release profile which make them a brilliant contender for many applications [24,61,62]. Recently, it has been debated that nanofibers should not only loaded with antimicrobial materials, but also should be combined with anti-inflammatory and tissue repairing materials [5,6,63].

Metronidazole (MTZ), a nitroimidazole derivative recognized for its activity as antiprotozoal, antiamebic and antibacterial drug [64,65,66,67,68]. MTZ antibiotic activity caused by rapid inhibition of anaerobic microbial cell replication of DNA. Additionally, it has anti-inflammatory and possible immunosuppressive properties that are likely to be promoted by its antioxidant activity and cell mediated immunity suppression. Moreover, the topical application of metronidazole has been reported to improve the wound healing process by secondary intention via improving differentiation of myo-fibroblasts in wounds [69,70,71].

Many researchers have reported the incorporation of metronidazole into electrospun nanofibrous composites for biomedical, tissue engineering, oral and dental applications as well as sustained release [72,73,74,75,76,77,78].

To the best of our knowledge, there was no study reporting the topical immobilization of metronidazole on PHB/Gel nanofibrous scaffold utilizing electrospinning technique to serve as a secondary intention wound healing accelerator and we are the first to report this.

Therefore, through the current research we are reporting the preparation of a novel nanofibrous composites based on poly-3-hydroxybutyrate (PHB)/gelatin (GeL)/metronidazole (MTZ) by electrospinning technique utilizing 2,2,2-tri fluoroethanol (TFE) as a fast volatilizing solvent.

Electrospun Nanofibrous composites were characterized by SEM, FTIR, TGA, water contact angel and mechanical properties. Additionally, electrospun nanofibrous composites PHB/Gel/MTZ were assessed in vitro for their cytocompatibilityon L-929 fibroblast utilizing CCK-8 assay, and in vivo manipulating animal rat models to evaluate the electrospun nanofibrous scaffolds as a candidate serving in wound healing by secondary intention and wound dressing applications. Moreover, antimicrobial activity for the nanofibrous composites against both Gram-positive and Gram-negative bacteria were evaluated histopathological and real-time PCR evaluations were also conducted for the wound.

## 2. Materials and Methods

### 2.1. Materials

Poly-3-hydroxybutyrate (PHB), gelatin (bovine skin, type B) (Gel), glutaraldehyde (GA) and dimethyl sulfoxide (DMSO) were purchased from Sigma-Aldrich (St Louis, MO, USA). 2,2,2-trifluoroethanol(TFE) and metronidazole (MTZ) were purchased from Acros-organics (Thermo Fisher Scientific, Waltham, MA, USA). The L-929 cell line was purchased from the Typical Culture Collection Committee Cell Bank, Chinese Academy of Sciences (Beijing, China). Cell counting kit-8(CCK-8)(2-(2-methoxy-4-nitrophenyl)-3-(4-Nitphenyl)-5-(2,4-disulfophenyl)-2H-tetrazole-monosodium salt) was procured from Beijing Solar bio-Science & Technology Co., Ltd., Beijing, China. Fetal bovine serum (FBS), Dulbecco’s phosphate-buffered saline (PBS) and Dulbecco’s modified Eagle’s medium (DMEM) were purchased from Gibco-BRL (Grand Island, NE, USA). All the materials and reagents were analytical grade and were used as received without further purification.

### 2.2. Animal and Ethical Approval

The in vivo cutaneous wound healing examination for the fabricated nanofibrous scaffolds were implemented on 21 adult’s male Wistar rats (9 weeks old; 180–200 g weight). The in vivo experiments protocols approved by the Research Ethical Committee of Institutional Animal Care and Use Committee at Alexandria University (ALEXU-IACUC) with approval number; AU14-200204-3-2. The rats were left for 7 days to be well adapted under ambient conditions (50–60% humidity, and 12 h light/dark cycle at 25 ± 2 °C) with free arrival to both standard rodent soft chow ad libitum and water.

### 2.3. Fabrication of Nanofibrous Scaffolds

**Scheme 1:**
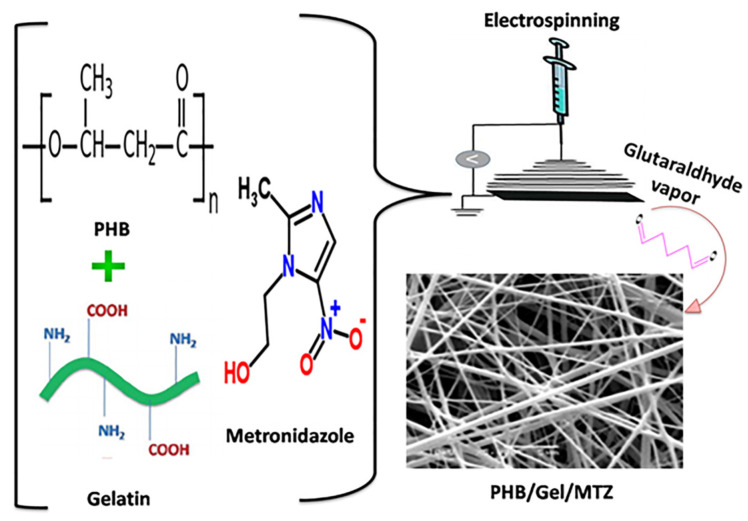
Schematic diagram of synthesis process and chemical structure of the molecules and polymers involved in the synthesis of nanofibrous scaffold Z_2_@7:3.

Polymer solutions were prepared according to Ramier et al. with some modifications [15]. Briefly, 0.35 g of PHB was dissolved in 5 mL TFE and gently stirred for 4 h at 50 °C, the concentration of PHB was 7% (*w*/*v*). Furthermore, 0.28 g PHB and 0.07 g Gel were completely dissolved in 5 mL TFE to obtain weight ratio of 8:2 (*w*/*w*) and gently stirred for 4 h at 50 °C, the polymer concentration of PHB/Gel was 7% (*w*/*v*). Additionally, 0.245 g PHB and 0.105 g Gel were completely dissolved in 5 mL TFE to obtain weight ratio of 7:3 (*w*/*w*) and gently stirred for 4 h at 50 °C, the polymer concentration of PHB/Gel was 7% (*w*/*v*). Subsequently, metronidazole was added to the as prepared polymer solutions at two different concentrations 5% (*w*/*w*) (Z_1_) and 10% (*w*/*w*) (Z_2_) based on total polymer solution concentration. Electrospinning device (NanoNC laboratory machine, South Korea Republic, ESR 100) was utilized to fabricate nanofibrous scaffolds. Concisely, the weight ratio (8:2 and 7:3) PHB/Gel/Z_1_ and weight ratio (8:2 and 7:3) PHB/Gel/Z_2_ solutions were filled into a 5 mL plastic syringe with a blunt-ended needle 23G (ID = 0.34 mm). An aluminum foil was used to collect nanofibrous scaffolds. The distance between the needle tip and the aluminum foil collector was 20 cm, dispensing rate conserved at 1 mL/h, and applied positive high voltage of 18 kV. The as-prepared nanofibrous scaffolds were placed in a vacuum drying oven at 40 °C for 2 days to remove the TFE remaining on the nanofibrous scaffolds till constant weight. The obtained nanofibrous scaffolds PHB/Gel at weight ratio (8:2 and 7:3) and loaded with two concentrations of MTZ weight ratio (8:2 and 7:3) PHB/Gel/Z_1_ and weight ratio (8:2 and 7:3) PHB/Gel/Z_2_ were stabilized against disintegration in biological fluids by placing in a sealed desiccator containing 10% (*w*/*w*) GA vapor for 48 h, subsequently placed in a vacuum drying oven at 40 °C for 2 days to eliminate unreacted GA residuals. Finally, the nanofibrous scaffolds were placed in a vacuum drying oven for subsequent testing and characterization. The schematic diagram of synthesis process and chemical structure of the molecules and polymers involved in the synthesis of nanofibrous scaffold Z_2_@7:3 are shown in Scheme 1.

### 2.4. Physical Characterization of Nanofibrous Scaffolds

The as prepared nanofibrous scaffolds were observed with a scanning electron microscope (SEM) (JEOL–JSM-6360LA, Tokyo, Japan) at an accelerating voltage of 10 kV. Nanofibrous scaffolds were sputter coated with gold before observed. The average diameters of nanofibers were measured with the help of Image analysis software (Image J, National Institute of Health, Bethesda, MD, USA), randomly, 100 nanofibers were selected from the SEM micrographs to determine nanofibers average diameters. Fourier transformer infrared spectrometer (FT-IR, Shimadzu FTIR-8400 S, Kyoto, Japan) was used to analyze composition and chemical structure of the nanofibrous scaffolds. The nanofibrous scaffolds were tested over a wavelength range of 4000–400 cm^−1^ to determine the present functional groups. A thermogravimetric analyzer (Shimadzu Thermal Gravimetric Analysis (TGA)-50, Kyoto, Japan) to analyze the thermal properties of the nanofibrous scaffolds via measuring their weight loss coinciding the gradual elevating of temperature from room temperature to 800 °C at a constant controlled heating rate of 10 °C/min under continuous nitrogen flow atmosphere.

### 2.5. Mechanical Properties of Nanofibrous Scaffolds

The tensile strength of the nanofibrous scaffolds was measured at ambient temperature using a universal testing Machine (Shimadzu UTM, Kyoto, Japan). Tensile measurements were carried out with cross heads movement at a constant speed of 5 mm/min under an automatic range. Tensile strength, elongation at break were calculated.

### 2.6. Water Uptake, Porosity and Contact Angles of Nanofibrous Scaffolds

The water uptake percentage of the nanofibrous scaffolds was measured based on study. The dry nanofibrous scaffold was weighed (*W*0), immersed in into phosphate buffer saline (PBS, pH = 7.4) at room temperature till equilibrium was reached. Subsequently, the surface wetness of the nanofibrous scaffolds was removed utilizing filter paper and wet scaffolds were weighed again (*Wi*). The following equation was used to calculate the equilibrium water uptake ratio.

(1) Water uptake (%)=(Wi−W0/W0)×100

where *W*0 and *Wi* is the initial and final weight of the samples, respectively.

The porosity of nanofibrous scaffold was measured according to liquid displacement method [79]. Briefly, a known weight of nanofibrous scaffold (*W*) was immersed in a divided up cylinder containing a known volume (*V*1) of absolute ethanol. After no bubbles found, the resulted volume was recorded (*V*2). The nanofibrous scaffold immersed in absolute ethanol was removed, and the residual volume of absolute ethanol was recorded (*V*3). The porosity of the nanofibrous scaffold was calculated according to the following equation.

(2) Porosity (%)=((V1−V3)/(V2−V3))×100

Contact angle meter VCA 2500 XE equipped with CCD camera and analysis software (AST Products, Billerica, MA, USA) were used to measure the contact angle. Where, a 0.03 mL of deionized water droplet was laid onto the surface of the nanofibrous scaffolds for 1 s, after that, images were captured using the attached camera.

### 2.7. Anti Bacterial Activity of Electrospun Nanofibrous Scaffolds

As an effective wound dressing, it is essential to maintain the sanitation of the wound bed site to sidestep the rise of bacterial strains that delay wound healing process, so the electrospun nanofibrous scaffold must have acceptable antimicrobial properties. In the present study antimicrobial activity of nanofibrous scaffolds PHB and PHB/Gel with different ratios (8:2 and 7:3) and nanofibrous scaffolds PHB/Gel containing MTZ at concentrations Z_1_ and Z_2_ as an antimicrobial drug was tested against Gram-negative and Gram-positive bacteria using disk agar diffusion technique [80]. Bacterial strain, *Escherichia coli* ATCC 10418 was chosen as a typical representative of Gram-negative bacteria and *Staphylococcus aureus* ATCC 6538 as a representative of Gram-positive bacteria, which were kindly provided from Genetic Engineering and Biotechnology Research Institute, City of Scientific Research and Technological Applications. Electrospun nanofibrous membranes were cut into disks (5 mm in diameter each). The disks of nanofibrous scaffolds were sterilized by UV-irradiation. Bacterial strains, *E. coli* and *S. aureus* were cultured into LB broth at 37 °C and shacked at 200 rpm for 24 h. One hundred µL of *E. coli* and *S. aureus* cultures (108 CFU mL^−1^) was spread onto LB agar plates. Subsequently, nanofibrous scaffolds discs (5 mm in diameter each) were placed onto agar surface. Plates were incubated at 37 °C for 24 h, after that, the diameter of the inhibition zone was measured in mm (inhibition zone is the diameter of the zone around the disc, together with the diameter of the disk). Experiments were conducted in triplicates (*n* = 3). Inhibition of strains growth can be considered effective through killing strains around, on the top and under the disk.

### 2.8. Release of MTZ from Electrospun Nanofibrous Scaffolds

The calibration curve of MTZ was created by detecting the absorbance values of metronidazole serial diluted concentrations at 310 nm. Briefly, two cassettes containing free MTZ and 10 mg of PHB/Gel/Z_2_ nanofibrous scaffold were immersed in 3 mL PBS buffer, and placed in a shaker incubator at 100 rpm, 37 °C. At predetermined time intervals, the buffer was withdrawn, and the same amount of fresh PBS buffer was added. The absorbance was detected at 310 nm with UV-spectrophotometer-double beam (T80+, PG instruments Ltd., England, UK). The MTZ contents in buffer were calculated and the percentage of MTZ released was calculated and cumulative release curve was illustrated.

### 2.9. Cell Seeding

L-929 mouse fibroblasts cell line were cultured in Dulbecco’s modified eagle’s medium (DMEM) containing 10% fetal bovine serum (FBS) and 1% antibiotic-antifungal agent in an atmosphere of 5% CO_2_ and 37 °C, and the medium was changed every other day. All samples of nanofibrous scaffolds (14 mm discs) were placed in 24-well plates and fixed with stainless steel rings. Before cell seeding, the nanofibrous scaffolds were sterilized under UV light for 12 h followed by washing with PBS 3 times, and then incubated with high glucose medium for 4 h to allow cell adhesion. L-929 cells were cultured at a density of 10,000 cells/wells.

### 2.10. Cytocompatibility of Nanofibrous Scaffolds

The viabilities assessment of L-929 cells proliferated on the nanofibrous scaffolds were assessed quantitatively by using CCK-8 assay on 1, 4 and 7 days. Subsequently, Cells were incubated in 10% (v/v) CCK-8 in DMEM at 37 °C for 2 h. The supernatants were collected and conveyed into 96 well plate for optical density measurements using a microplate reader (Multiskan MK3, Thermo Fisher, Waltham, MA, USA) at 450 nm (*n* = 3).

### 2.11. In Vivo Wound Healing Study

The in vivo cutaneous wound healing examination for the fabricated nanofibrous scaffolds were implemented on 21 adult’s male Wistar rats (9 weeks old; 180–200 g weight). The in vivo experiments protocols approved by the Research Ethical Committee of Institutional Animal Care and Use Committee at Alexandria University (ALEXU-IACUC) with approval number; AU14-200204-3-2. The rats were left for 7 days to be well adapted under ambient conditions (50–60% humidity, and 12 h light/dark cycle at 25 ± 2 °C) with free arrival to both standard rodent soft chow ad libitum and water. The rats were divided into three groups (*n* = 7/group) at which group one (negative control): received sterile gauze, group two (positive control): received blank nanofibrous scaffold (PHB/Gel) with ratio 7:3, and group three: received nanofibrous scaffold (PHB/Gel) with ratio 7:3 containing MTZ at concentration of Z_2_ (Z_2_@7:3). After anesthesia with intramuscular injection of 10% Ketamine Hydrochloride “(Dopalen^®^, 0.1 mL/100 g body weight)” and 2%xylazine hydrochloride “(Calmium^®^, 0.1 mL/100 g body weight)”, the dorsal hair was shaved via an electric animal razor followed by sterilization of the skin using 70% ethanol solution and chlorhexidine. After shaving and sterilization a deep full-thickness circular excisional wound of 1.5 cm diameter was prepared with the help of sterile surgical scissors, Surgical scalpel, and forceps on the center of the hairless skin [81,82,83]. The nanofibrous scaffolds including blank and Z_2_@7:3 were applied topically on the wound area every two days to investigate their potential application in wound healing compared to untreated wound (negative control) receiving sterile gauze. The wound area was cleaned with saline before applying the treatment then, fixed with elastic adhesive bands for adequate fixation of the applied nanofibrous scaffold. The macroscopic examination of the wound closure was observed at day 3, 7, 10 and 14 immediately after operation using digital camera. At both days 7 and 14 the wound tissue sample was removed from the rats after sacrificing using high dose of anesthesia followed by cervical dislocation for histopathological examination. The excised skin was fixed in 10% formalin, stained with hematoxylin and eosin (H&E) for estimation of epithelialization and keratinization and Masson’s trichrome stainings (MTS) for estimation of collagen deposition under an optical microscope [84,85,86].The wound area was calculated with the help of digital caliber and the % contraction of the wound area was calculated using the following equation [87].

(3) Wound closure (%)=1−(Wound area at (n day)/Wound area at (0 day))×100

### 2.12. Gene Expression of Interleukin-6 (IL-6) and Transforming Growth Factor-β1 (TGF-β1)

The skin tissue from different treated groups was accurately transferred into RNase-free tube. The thawed skin tissues were then homogenized using 1 mL sample lysis buffer then the total RNA was isolated from the skin tissues using BioFlux simply P total RNA extraction kit, cat. # BSC52S1 (Bioer, Zhejiang, China) according to the instructions supplied by the manufacturer. Both the concentration and the purity of the isolated RNA was assessed using a NanoDrop™ UV-Vis spectrophotometer (Nanodrop Technologies Thermo Scientific, Inc., Waltham, MA, USA). The complementary DNA (cDNA) was created by using the reverse transcription kit cat. # K1621 (Thermo Fisher Scientific, Inc., Waltham, MA, USA) according to the instructions supplied by the manufacturer. Then, the amplification reactions for the cDNA were completed via Maxima SYBR green qPCR Master Mix (2X), cat. # K0251 (Thermo Fisher Scientific, Inc., Waltham, MA, USA) with 2.5 μL of cDNA and 1.5 μL of both transforming growth factor-β1 (TGF-β1) and interleukin-6 (IL-6) primer then the volume of the reaction mixture was completed to 25 μL using QIAGEN Rotor-Gene Q real-time PCR thermal cycler, according to the instructions supplied by the manufacturer (1 cycle of initial denaturation at 95 °C for 10 min followed by 40 cycles of 15 s at 95 °C, 30 s at 57 °C and 30 s at 72 °C). The primer sequences utilized for cDNA amplification were as follows: for IL-6 the forward sequence was (5-TTTCTCTCCGCAAGAGACTTCC-3) and the reverse sequence was (5-TGTGGGTGGTATCCTCTGTGA-3); for TGF-β1 the forward sequence was (5-TGACATGAACCGACCCTTCC-3) and the reverse sequence was (5-TGTGGAGCTGAAGCAGTAGT-3). The level of β-actin expression as a house keeping gene was applied as an internal control for each specimen. The forward and reverse primer sequences for β-actin were (5-AGATCAAGATCATTGCTCCTCCT-3) and (5-ACGCAGCTCAGTAACAGTCC-3), respectively. The delta comparative Ct (2^−ΔΔCt^) analysis was applied for the assessment of the level of changes in gene expression. The melting curve analyses were completed for all amplifications to confirm that only single products were created from the reactions. The oligonucleotide sequence of the primers were fabricated by Willowfort, Birm, UK [88].

### 2.13. Statistical Analysis

Costate software was used to analyze the obtained data statistically. The one-way ANOVA was used, followed by the LSD test for multiple comparisons. The data were expressed as the mean ± standard deviation (SD). In all evaluations, *p* < 0.05 was considered to be statistically significant.

## 3. Results and Discussions

**Figure 1:**
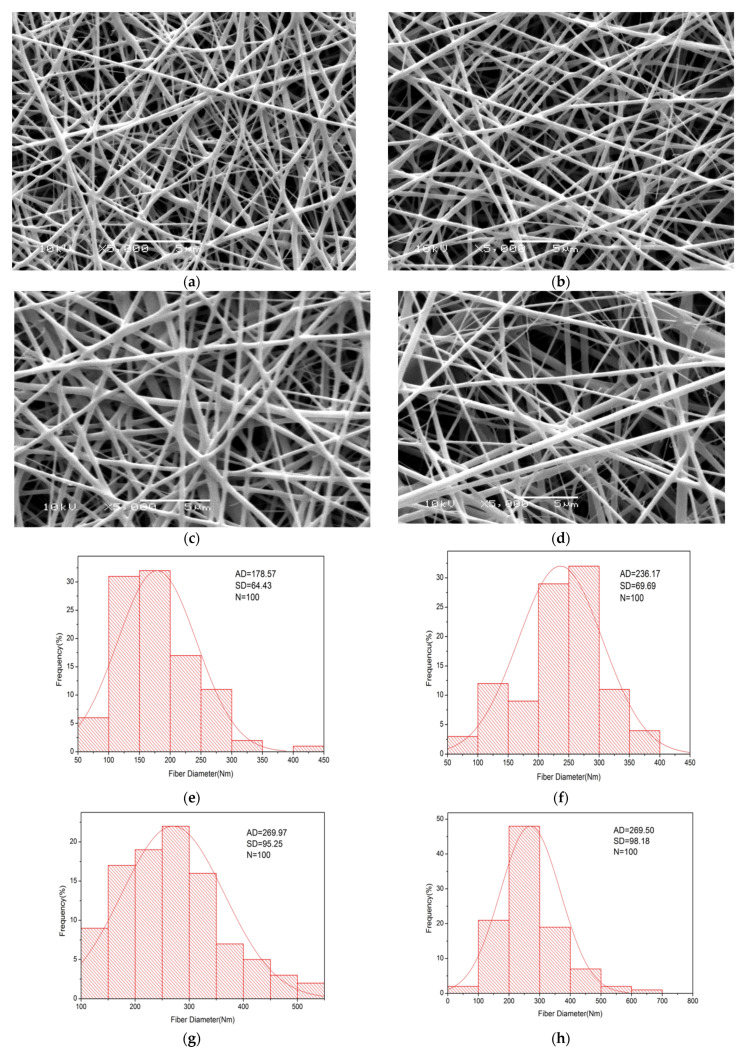
Nanofibrous scaffolds morphology and their relative histogram exhibiting average fiber diameter. SEM images of PHB/Gel nanofibrous scaffolds with different ratios (8:2 and 7:3) loaded with two different concentrations of MTZ 5% (Z_1_) (**a**,**c**) and MTZ 10% (Z_2_) (**b**,**d**), respectively, and histograms exhibiting average fiber diameter with SD± (**e**–**h**).

The micro-structure and morphology of the nanofibrous scaffolds were detected under high magnifications and their average diameters are shown in Figure 1. Uniform, ultrafine, smooth, continuous and beadless nanofibrous scaffolds with porous 3D structure and no drug accumulates on the surface were successfully fabricated after several optimizations by electrospinning PHB/Gel of nanofibrous scaffolds with different ratios (8:2 and 7:3), respectively, loading content of 5 wt% and 10 wt% MTZ as Z_1_ and Z_2_, respectively. The average diameter of the nanofibrous scaffolds were 178.57 ± 64.43 nm and 269.97 ± 95.25 nm for PHB/Gel with ratios (8:2 and 7:3) containing MTZ with concentration of 5% (Z_1_), and 236.17 ± 70 nm and 269.5 ± 98.18 nm for PHB/Gel with ratios (8:2 and 7:3) containing MTZ with concentration of 10% (Z_2_), respectively, as shown in Figure 1. SEM images of the prepared PHB, PHB/Gel with different ratios (8:2 and 7:3) were shown in the Supplementary Material File (Figures S1–S3).

**Figure 2:**
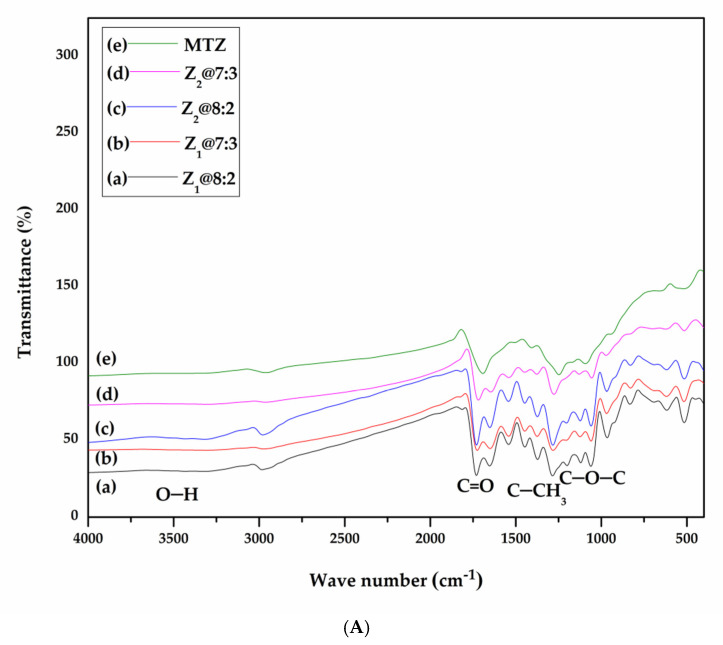

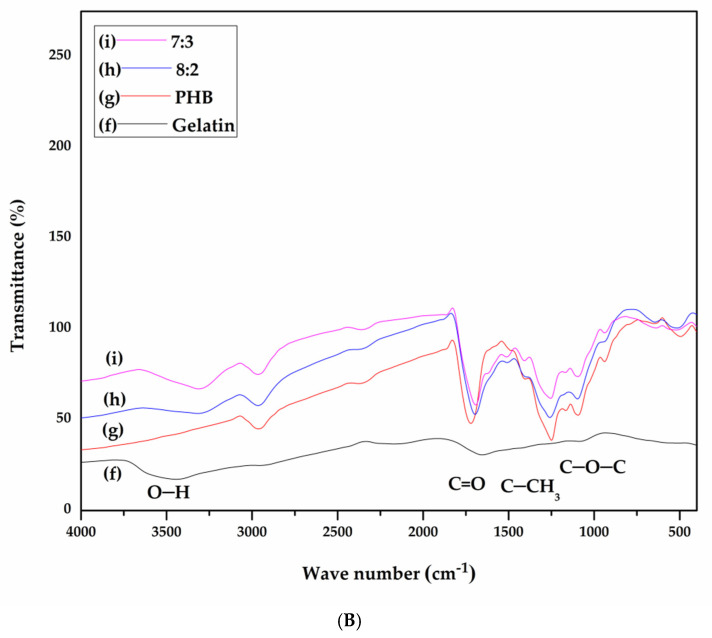
FT-IR spectra of (**A**) MTZ, PHB/Gel with different ratios loaded with MTZ at two different concentrations Z_1_ and Z_2_.; (**B**) gelatin, PHB and PHB/Gel with different ratios (8:2, 7:3).

FT-IR was utilized to recognize characteristic peaks and foremost changed appearing within polymers blends. The infrared absorption spectra of metronidazole (MTZ) drug, the PHB/Gel based nanofibrous scaffolds, with different weight composition (8:2 and 7:3; *w*/*w*), and finally the PHB/Gel based nanofibrous scaffolds incorporated with two different concentrations of MTZ Z_1_ and Z_2_. In Figure 2, infrared absorption spectrum revealed MTZ characteristic peaks (O–H stretching)at 3414 cm^−1^ and 3220 cm^−1^, (=CH stretching) at 3097 cm^−1^ and (–NO_2_ antisymmetric stretching) at 1550 cm^−1^ [72]. Infrared absorption spectra of PHB/Gel with different ratios (8:2 and 7:3) were compared with and without MTZ (Z_1_ and Z_2_). For PHB, the presence of acidic –OH absorption band is observed at 3440 cm^−1^, characteristic peaks of the carbonyl group C=O stretching vibration band is observed at 1725 cm^−1^. Peaks observed at 973, 1293 and 1725 cm^−1^ are due to the crystalline phase of PHB and peak at 1186 cm^−1^ resulting from PHB amorphous phase. The C–O stretching band at 1055 cm^−1^ and the –CH– bands at 2970 cm^−1^ [15,33]. For Gel, FT-IR reveals the presence of characteristic peaks of Gel functional groups as, N–H stretching at 3306 cm^−1^, implying the presence of amine function and the amide bands peaks at 1650 cm^−1^ (Amide I), 1540 cm^−1^ (AmideII) [72,89].

**Figure 3:**
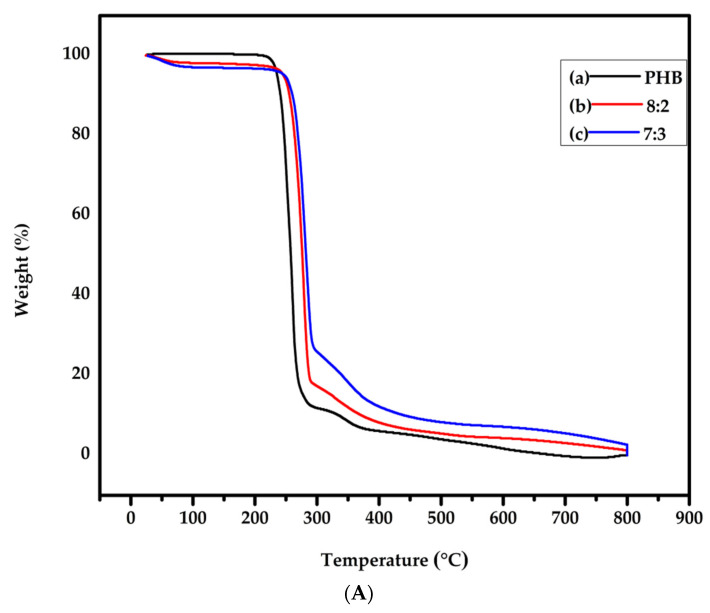

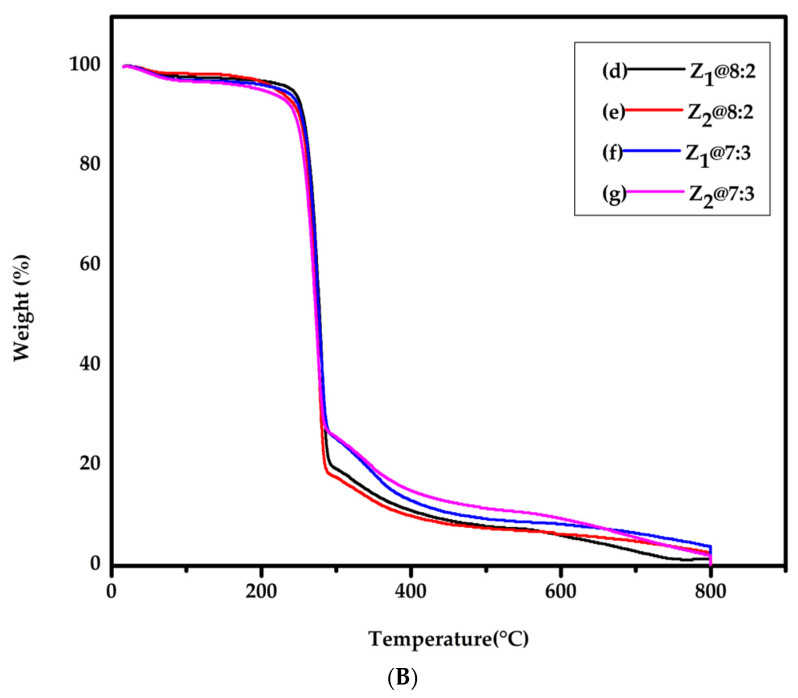
TGA curves of (**A**) PHB and PHB/Gel with different ratios (8:2 and 7:3); (**B**) PHB/gel/MTZ (8:2 and 7:3) with two different concentrations Z_1_ and Z_2_.

TGA measurement were conducted for nanofibrous scaffolds seven groups, where TGA thermographs were illustrated in Figure 3, while the thermal data were recapitulated in Table 1. All samples showed several decomposition steps due to their diverse component. The obtained results showed that in all samples a slight weight loss at temperature range ≤ 100 °C was observed, which is related to removal of moisture and or residual solvent. TGA graphs verified that nanofibrous scaffolds containing MTZ (Z_1_@8:2, Z_1_@7:3, Z_2_@8:2 and Z_2_@7:3) has Tonset values (243.36, 231.36, 223.75 and 209.27 °C), respectively, for nanofibrous scaffolds without MTZ (8:2 and 7:3) Tonset values at (246.11 and 247.91 °C), respectively. Moreover, 50% weight loss temperature Tmax values of nanofibrous scaffolds containing MTZ (Z_1_@8:2, Z_1_@7:3, Z_2_@8:2 and Z_2_@7:3) were (277.76, 276.97, 273.59 and 272.56 °C), respectively, for nanofibrous scaffolds without MTZ (8:2 and 7:3) Tmax values were (276.18 and 282.14 °C), respectively. The results illustrated that the addition of Gel and incorporation of MTZ to PHB caused a small thermal decomposition shifting to a lower temperatures. Generally, the as prepared nanofibrous scaffolds were appropriate to be applied in biomedical applications.

Nanofibrous scaffold for wound healing application should embrace appropriate mechanical properties to assure facile management on the wound bed site. Tensile strength and elongation at break (%) of nanofibrous scaffolds seven groups were assessed to estimate the performance of it under mechanical loads, where results are summarized at Table 2. The nanofibrous scaffolds without MTZ incorporation showed high tensile strength and high elongation at break compared with the nanofibrous scaffolds incorporated with MTZ. The decrease in tensile strength can be endorsed to some interaction between nanofibrous scaffold matrix after incorporation of MTZ. Therefore, the mechanical strength of nanofibrous scaffolds incorporated with MTZ were intensely lowered.

**Figure 4:**
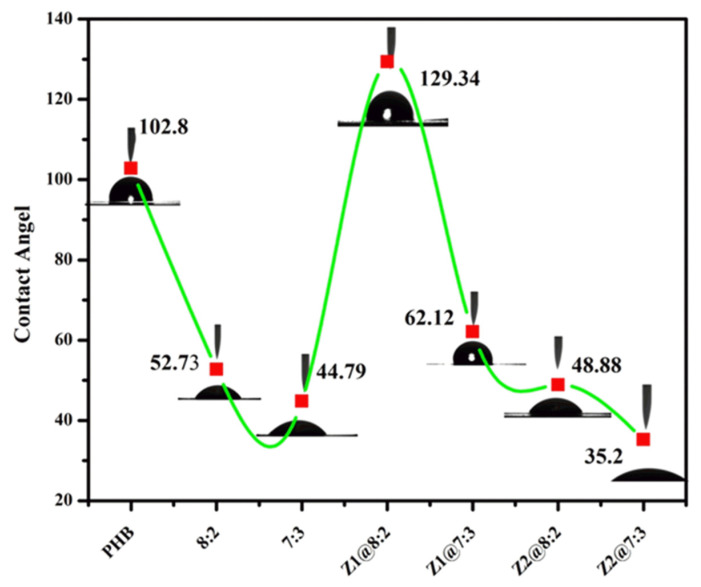
Contact angle values of nanofibrous scaffolds PHB, 8:2, 7:3, Z_1_@8:2, Z_1_@7:3, Z_2_@8:2 and Z_2_@7:3.

The hydrophilicity of nanofibrous scaffold has a great influence on cell proliferation and adhesion. Generally, the water contact angle of the PHB is 102.8° while the water contact angle of 52.73° and 44.79° for PHB/Gel 8:2 and 7:3, respectively. While the contact angles 129.34° and 62.12° for Z_1_@8:2 and Z_1_@7:3, respectively. Additionally, the contact angles 48.88° and 35.20° for Z_2_@8:2 and Z_2_@7:3, respectively, as shown in Figure 4. Because of the amine and carboxyl functional groups in the gelatin structure, gelatin considerably improves the hydrophilicity of the membranes. Because of the hydroxyl and polar imidazole ring functional groups on the MTZ molecule, the contact angle decreases as the MTZ content rises. The rate of tissue regeneration and membrane biodegradation will both rise as the hydrophilicity increases [72]. Moreover, our findings agrees with the results reported by Sanhueza et al., which indicates that gelatin containing scaffolds has contact angle lower than PHB scaffold [90].

Water uptake study of the as prepared nanofibrous scaffolds is ruminated one of basic considerations for biomaterials, hence nanofibrous scaffold is designed to be utilized in a moist environment to absorb excess exudates. To such an extent, pore diameter is inversely proportional to gelatin fraction in the scaffold, and pore size should grow as water uptake increases. However, as being reported by Nagiah et al., because of the crosslinking process, which causes an increase in fiber diameter and a decrease in pore size, the water uptake behavior of scaffolds does not reach its maximum apex [33]. Moreover, the ratio of Gel and amount of MTZ incorporated both have direct consequence on water uptake and porosity of nanofibrous scaffolds as shown in Table 2. Moreover, porosities of PHB/Gel/MTZ nanofibrous scaffolds with different ratios (8:2 and 7:3) loaded with MTZ at two different concentrations Z_1_ and Z_2_ showed values lied in the range of 60–70% which is desired for tissue engineering applications as reported by Chong et al. [91]. It has also been reported by Shan et al. that the porosity around 87% for SF/Gel nanofibrous composite is appropriate for wound dressing applications [92].

**Figure 5:**
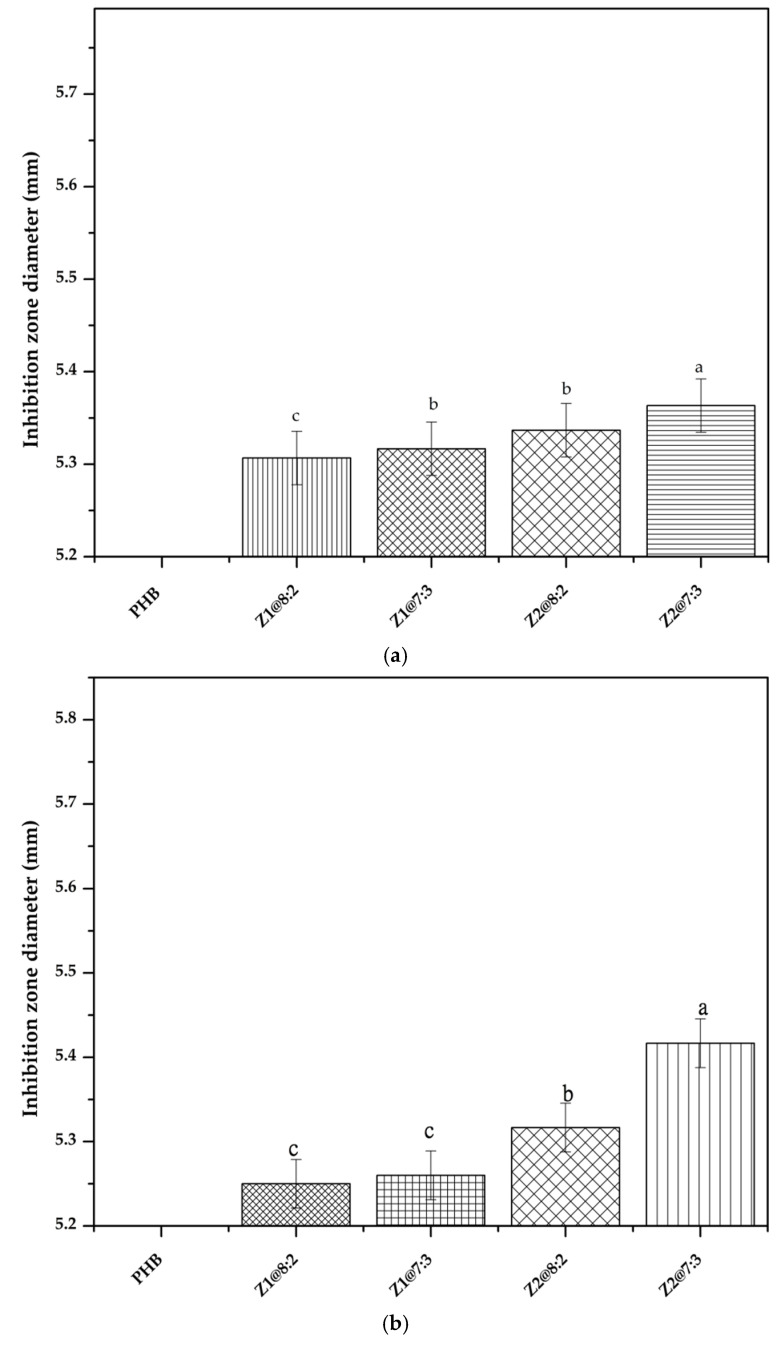
Antimicrobial activity of nanofibrous scaffolds against (**a**) *E. coli*. (**b**) *S. aureus*. Different letters mean statistically significant differences for a *p*-value < 0.05.

Several favorable characteristics of nanofibers such as a large surface area-to-volume ratio, flexibility in surface functionalities and superior mechanical performance, which facilitated utilization of nanofibers in antimicrobial drug delivery in various fields, such as tissue engineering, wound dressing and medical implants [93]. Antimicrobial activity of nanofibrous scaffolds PHB, PHB/Gel prepared in ratio of 8:2 and 7:3 and PHB/Gel loaded with MTZ (5% and 10%) were tested against Gram-negative bacteria, *E. coli*. Nanofibrous scaffolds Z_1_@8:2 and Z_2_@7:3 were the highest against *E. coli* with 5.43 mm and 5.33 mm, respectively. On the other hand, Z_1_@7:3 and Z_2_@8:2 had the lowest inhibition zone diameter to reach 5.3 mm with no significant difference between them Figure 5. Furthermore, antimicrobial activity of nanofibrous scaffolds PHB, PHB/Gel prepared in ratio of 8:2 and 7:3, and PHB/Gel loaded with MTZ (5% and 10%) were tested against Gram-positive bacteria, *S. aureus*. The data obtained in the current study showed nanofibrous scaffoldZ_2_@7:3 had the highest significant effect in inhibiting the bacterial growth of *S. aureus*. The diameter of inhibition zone reached its maximum value to reach 5.42 mm Figure 5. Nanofiber poly-(ε-caprolactone) (PCL) and gelatin blended with metronidazole (MTZ) content over 5% showed observable antibacterial activity (Xue et al., 2014) [64,72]. Moreover, El-Newehy et al. (2012) reported that electrospun poly-(vinyl alcohol) (PVA)/poly(ethylene oxide) (PEO) nanofiber loaded with metronidazole (MTZ) as an antimicrobial drug showed a significant antimicrobial activity against Gram-negative bacteria.

**Figure 6:**
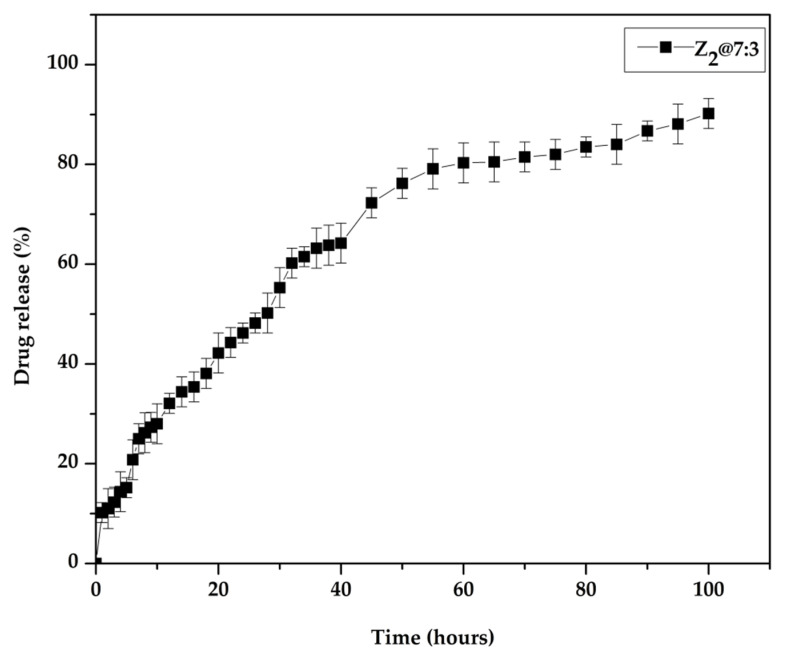
In vitro release profile of MTZ from Z_2_@7:3 nanofibrous scaffold in PBS (pH = 7.4).

In vitro drug release behavior of MTZ was assessed in phosphate buffered saline solution. The percentage of MTZ released from the nanofibrous scaffold released is shown in Figure 6. The percentage of MTZ loading content was determined prior to the release study to calculate the percentage of release from nanofibrous scaffold Z_2_@7:3. MTZ release from nanofibrous scaffold Z_2_@7:3 started with initial burst release profile, that can be attributed to hydroxyl and polar imidazole ring functional groups on the MTZ accumulated on the surface of the nanofibrous scaffold followed by sustained release profile up to 100 h. Jeyakumar et al., reported that PHB/Gel/silver sulfadiazine nanofibrous matrix acts as carrier to deliver SSD at the wound site in a controlled manner up to 80 h [94]. Additionally, it has been reported by Hu et al., that polymers with higher crystallinity may be responsible for faster release profiles [95].

**Figure 7:**
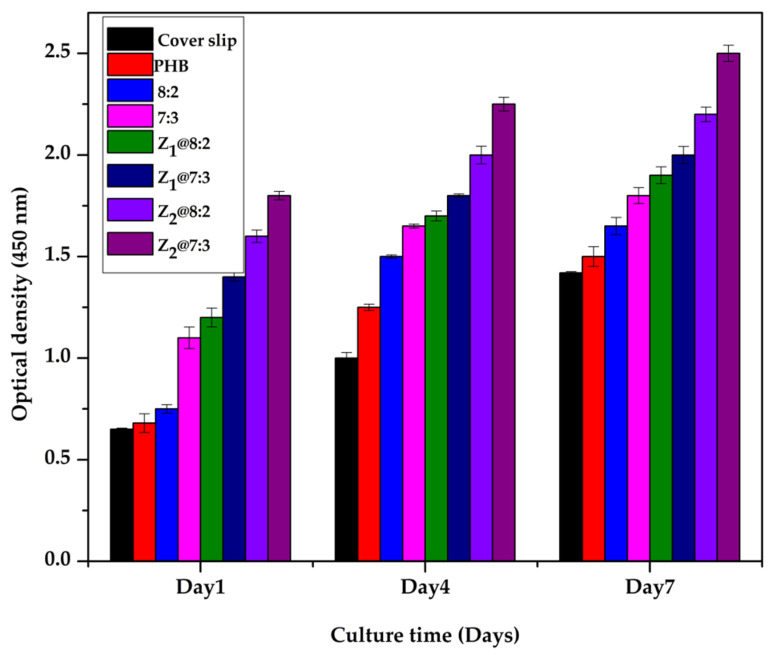
Cell biocompatibility on different nanofibrous scaffolds. L-929 cells proliferation on PHB/Gel/MTZ nanofibrous scaffolds.

Cytocompatibility of nanofibrous scaffolds were assessed by CCK-8 assay Figure 7. L-929 fibroblasts showed proliferation from day 1 to day 7. At day 4, the number of cells proliferated on nanofibrous scaffolds incorporated with MTZ was higher than the ones without MTZ and the cover slip, which is statistically significant. The constant release of MTZ from nanofibrous scaffold augmented the proliferation of L-929 cells. Moreover, at day 7 cells on nanofibrous scaffold Z_2_@7:3 grew fast compared with other nanofibrous scaffolds and the cover slip. Depending on these results, we postulate that nanofibrous scaffold Z_2_@7:3 is nontoxic and augment cell proliferation and differentiation, and would be serving as a suitable candidate for skin tissue regeneration. Naderi et al., reported that cell growth and morphology was affected by the hydrophobic behavior of PHB nanofibers [96]. Moreover, Trindade et al., reported that topical application of 4% metronidazole solution had a direct action on fibroblasts via activating the development of keratinocytes and stimulating the reepithelialization [69]. Additionally, Xue et al. reported that cells could proliferate on PCL/gelatin nanofibrous membranes without cytotoxicity till 30% MTZ concentration [72].

**Figure 8:**
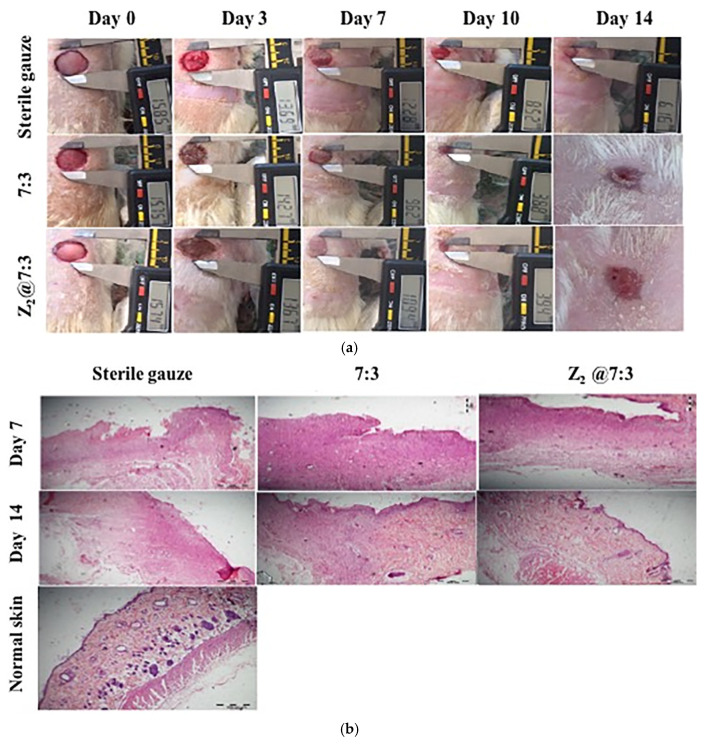

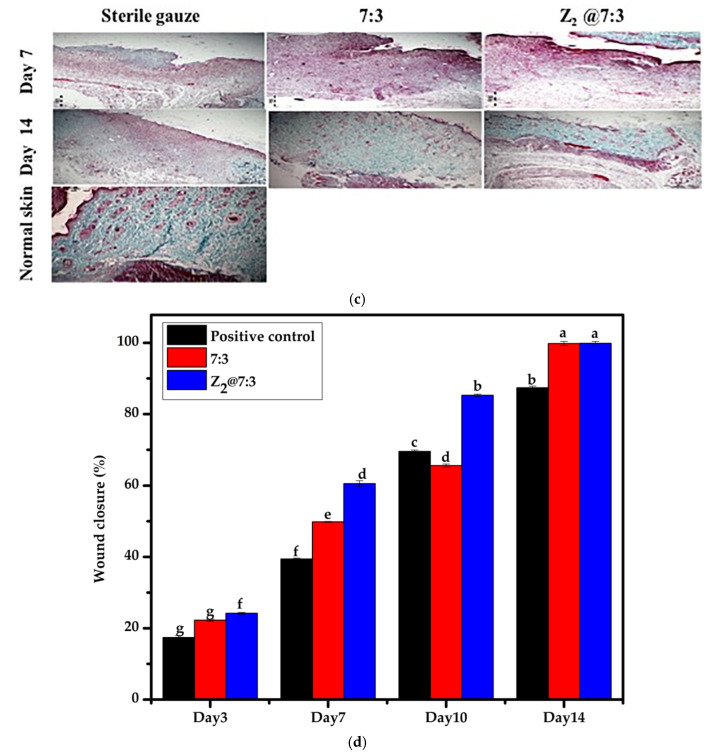
(**a**) Photographic images of in vivo full thickness excision wounds after treatment with sterile gauze, blank and Z_2_@7:3 for 14 days. (**b**) Histological analysis of treated wounds on days 7 and 14 using H&E stain. (**c**) Histological analysis of treated wounds using Masson’s trichrome Stain (MTS) on day 7 and 14 (original magnification = 100). (**d**) Percentage of wound closure in three different groups at different time, different letters mean statistically significant differences for a *p*-value < 0.05.

The efficiency of fabricated nanofibrous scaffolds compared to sterile gauze for wound healing applications was assessed via full thickness wound model. Topical delivery of the antimicrobial agents leads to accumulation of higher drug concentrations at the target site, and so, reduced the side effects associated with dose dose-related systemic toxicity and evolution of the bacterial resistance. Metronidazole is a potent antimicrobial agent suitable for local administration with a higher efficiency against anaerobic bacteria and some pathogenic protozoa [97]. Topical delivery of metronidazole via incorporation into electrospun nanofibers is an efficient method to achieve a sustained drug release behavior at the wound site. The fabricated PHB/Gel/MTZ nanofibrous scaffold (Z_2_@7:3) is effective in producing a proper platform in the surface of the wound via inhibiting the microbial growth and encouraging the intercellular reaction for the formation of fresh granulation tissue. The photographic images of the wound area after treatment with nanofibrous scaffold PHB/Gel (7:3) and the calculated % wound closure rates are displayed in Figure 8 and Table 3, respectively. At the end of the experiment (14 days), rats applied sterile gauze displayed a low advance in the wound healing with 87.39% wound closure, while rats applied PHB/Gel (7:3) and PHB/Gel/MTZ(Z_2_@7:3) nanofibrous scaffolds showed 99.79% and 99.83% wound closure, respectively. All the rats treated with PHB/Gel nanofibrous scaffolds either free or loaded with MTZ displayed highly effective wound healing and complete wound closure with normal granulation tissue and no exudate at day 14.

The histological features of the treated skin sections from different groups compared to normal skin and stained with hematoxylin and eosin (H&E) and Masson’s trichrome (MTS) are illustrated in Figure 8b,c on days 7 and 14. The histopathological examination was centralized on the wound healing ability of rat tissue by using the fabricated nanofiber. The normal skin image presented the typical structural elements of the healthy skin such as intact epidermis, dermis (connective tissue layer), muscles, sebaceous glands, blood vessels and hair follicles as explained in Figure 8a. The complete healing and epithelization were recorded for the groups treated with PHB/Gel nanofibrous scaffold loaded with MTZ (Z_2_@7:3) and blank PHB/Gel (7:3) nanofibrous scaffold. The H&E stain image on the 7th day showed that the sterile gauze treated group displayed a great ulcer associated with a high number of inflammatory cells without any obvious reepithelialization associated with the lack of both sebaceous glands and intact hair follicles. While on 14th day the ulcer got smaller at the wounded area associated with a high number of inflammatory cells without any obvious reepithelialization associated with the lack of both sebaceous glands and intact hair follicles. On the other hand, at 7th day the skin wound area from groups treated with PHB/Gel nanofibrous scaffold loaded MTZ (Z_2_@7:3) and blank PHB/Gel (7:3) nanofibrous scaffold showed no ulcer with a moderate reepithelialization and less inflammatory response, and reconstructed dermal layer with the beginning of the appearance of new skin supplements including hair follicle and sebaceous glands. While on 14th day the treated groups displayed the features of the normal skin, like a well created epidermal and dermal layers loaded with a massive connective tissue, hair follicle (HF), sebaceous gland (SG) and blood vessels (BV). At the end of the experiment, a full sealed skin at the damaged site was observed for the groups treated with the fabricated nanofiber. Previous surveys proved the substantial functions performed by the angiogenesis in the restoration of both epidermis and dermis containing hair follicles [98,99]. The consistency of collagen deposition and maturation at the wound site were rated among different treated rats’ groups via Masson’s trichrome staining (MTS) Figure 8c. Dense and intensive deposition of collagen fibers is a substantial phase to guarantee perfect wound healing. Collagen fibers were spotted as blue green color. The more the density of the stain, the more the amount of collagen fiber deposited [81].

The histopathological image of MTS for the normal skin section exhibited uniform, mature, intensive and normal collagen fiber deposition as explained in Figure 8c. On the 7th and 14th day, the group treated with sterile gauze exhibited very much less, collagen deposition on the dermal layer compared to the normal skin and other treated groups. The group treated with blank PHB/Gel (7:3) nanofibrous scaffold exhibited a modest collagen fiber deposition in the dermal layer on the 7th day which then becoming tighter and mature on the 14th day. On the other hand, the group treated with PHB/Gel nanofibrous scaffold loaded MTZ (Z_2_@7:3) exhibited a modest collagen fiber deposition in the dermal layer on the 7th day which then become more intensive, integrated and matured collagen fiber deposit parallel to the epidermis at the 14th day and mimic the collagen fiber deposition on normal skin. The skin regeneration potential for PHB/Gel (7:3) nanofibrous scaffold was formerly evidenced by Nagia et al. [33]. The nanofibrous scaffold confirming the fast proliferation of human dermal fibroblasts and keratinocytes with common morphology. The complete highly effective wound closure with an excellent attachment of fibroblast was formerly reported for PHB/Gel/collagen electrospun nanofiber loaded with ostholamide (OSA) as an antimicrobial drug during 15 days compared to the control group that took more than 20 days for complete healing [6,100].

The highly effective antimicrobial activity of metronidazole boost its potential application as a valuable topical drug for enhancing the quality of wound healing [101].

**Figure 9:**
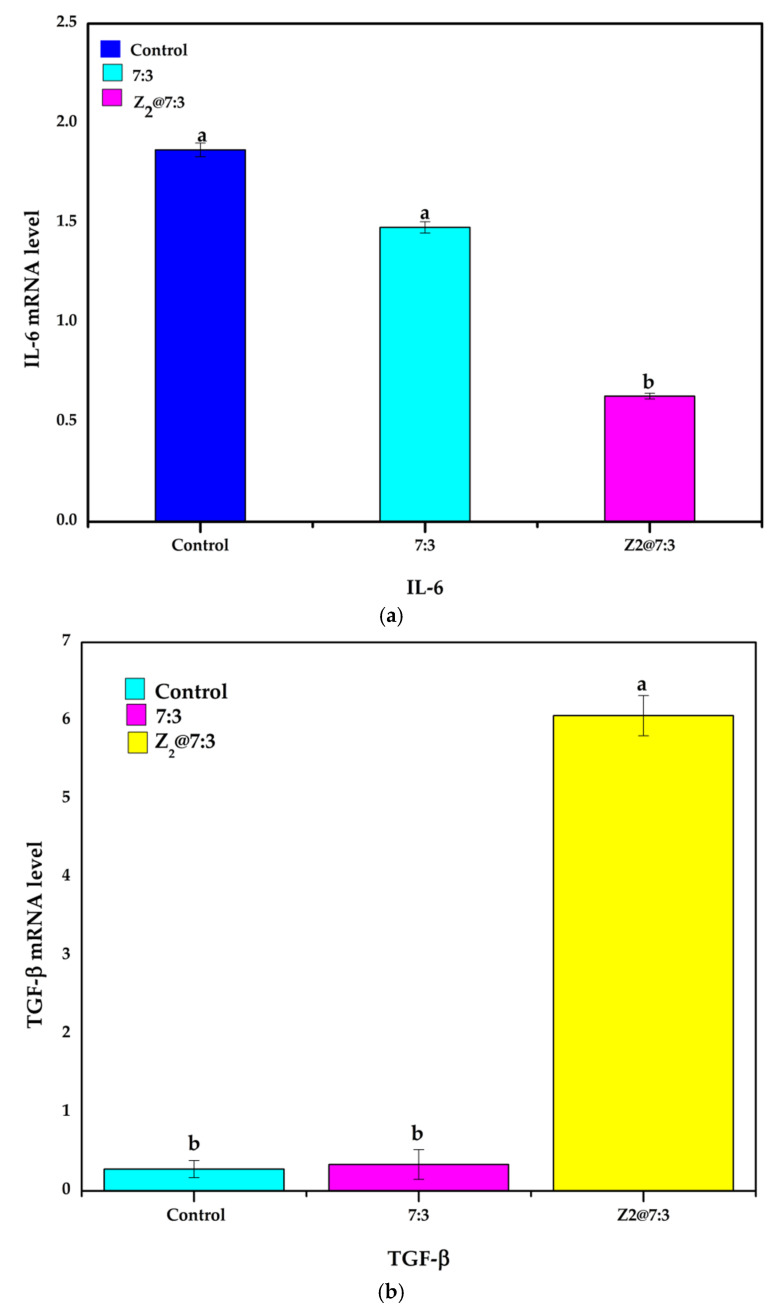
Expression levels of genes related to wound healing on day 14. (**a**) IL-6; (**b**) TGF-β. Different letters mean statistically significant differences for a *p*-value < 0.05.

The level of the gene expression of both TGF-β1 and IL-6 were assessed for further investigation of the exact mechanism of the fabricated nanofibers on wound healing. Transforming growth factor β1 (TGF-β1) is located in the injured site through platelet degranulation subsidiary to tissue damage and has a primary role in the handling of healing process. Studying the expression level of TGF-β1 is substantial due to its greater influence on the healing steps via excessive formation of granulation tissue in the wounded area, repressing the inflammation and maximizing both type I and II collagen generation [102,103]. It is also a potent metalloproteinase suppressor, thus preventing collagen breakdown. It was manifested post injury and have raised gradually by time till complete healing. Moreover, TGF-β encourage endothelial cell migration and angiogenesis besides its pivotal role in the wound contraction and closure via encouraging the differentiation of fibroblasts into myofibroblasts [104]. Moreover, TGF-β can subside superoxide generation from macrophages which helps to conserve surrounding healthy tissue and equipped the wound for generation pf granulation tissue [105]. As shown in Figure 9b, the level of gene expression for TGF-β1 in the wounds topically treated with nanofiber loaded with metronidazole was significantly enhanced (*p* < 0.05) in comparison to untreated wounds that applied sterile gauze and blank nanofiber. The ultimate obvious enhancement in the level of TGF-β1 expression level was spotted for nanofiber loaded with metronidazole with about 5.731-fold increase in its level of expression compared to wounds treated with blank nanofiber and sterile gauze which exhibited 1.286 and 0.300-fold increase in the level of TGF-β1 expression, respectively. Based on the aforementioned actions, which elucidate the primary role of this growth factor in the cutaneous wound healing procedure through acceleration of granulation tissue synthesis accompanied with maximum collagen deposition. So, topical application of nanofiber loaded with metronidazole could promote the skin wound healing via up-regulation of TGF-β1 gene expression level. The higher expression level of TGF-β1 for metronidazole loaded nanofiber is mainly attributed to the previously reported ability of metronidazole to accelerate the healing via encouraging the angiogenesis and production of collagen in second intention wounds [106]. The maximum reepithelialization and faster corneal haling was previously reported for corneal ulcer after treating rats cornea with 0.1% metronidazole ophthalmic solution for seven days after injury. The authors concluded that metronidazole accelerate the corneal healing via modulating tissue angiogenesis as it has proangiogenic action [107,108]. It was previously reported by Trindade and his colleagues that topical application of 4% metronidazole solution had a direct action on fibroblasts via activating the development of keratinocytes and stimulating the reepithelialization and wound healing on the third day [69].

Inflammation is a normal physiological state which appear in the early stage of wound. Inflammation can maintain the wound from microbial attack and eliminate dead tissues via its substantial function in the organization of both hemostasis and innate immune system. Protracted inflammation can interfere and impair with the normal phases of wound healing leading to a decline in the activation and differentiation of keratinocytes accompanied with exaggerated scare formation [109]. On contrary to TGF-β tumor necrosis factor (TNF) α is detected early in wounds after injury and decreased gradually by time till complete healing [103]. The nonhealing wounds display a higher matrix metalloproteinase (MMP) activity, collagenases and gelatinases level generated by (TNF) α and IL-1β that degrades the extracellular matrix (ECM) and suppresses cell migration and collagen deposition [105].

Interleukin-6 (IL-6) is a pro-inflammatory cytokine generated by resident macrophages and infiltrating monocytes via exposure to wound lesions. The macrophages are then transformed from M1 pro-inflammatory phenotype to M2 tissue repairing phenotype accompanied with expression of anti-inflammatory mediators [109,110]. The level of the expression of IL-6 gene was significantly reduced (*p* < 0.05) in the wounds topically treated with nanofiber loaded with metronidazole in comparison to untreated wounds that applied sterile gauze and blank nanofiber Figure 9a. The obvious reduction in the level of IL-6 which is about 4.6-fold compared to sterile gauze and 1.4-fold compared to blank nanofiber is mainly due to the previously reported anti-inflammatory activity of metronidazole [111,112].

The clinical potential of metronidazole ointments at a concentrations (1–4%) in the treatment of skin inflammatory diseases such as atopic dermatitis AD was previously evidenced by Nishimuta et al. [113]. In another study the in vivo anti-inflammatory activity of metronidazole has been reported in combination with palmitoleic acid via lowering the free radicals created by neutrophil at the inflammation sites in the skin and suppress oxidative tissue damage [114]. The anti-inflammatory activity of metronidazole in lowering the free radicals created by neutrophils might be implicated in its effects on rosacea [115]. In conclusion, the topical application of metronidazole has been reported to improve the healing via combination of three mechanisms including: repression of the inflammatory reaction so minimizing tissue eradication, progression of peripheral wound epithelialization and growth of keratinocytes, and lowering the bacterial load [112].

## 4. Conclusions

A novel multifunctional nanofibrous scaffold was prepared utilizing electrospinning technique based on PHB, Gel and MTZ. The results illustrate that PHB/Gel/MTZ nanofibrous scaffolds has respectable hydrophilicity, porosity and water uptake. Furthermore, the incorporation of MTZ exhibited good mechanical and thermal properties, and, also, augment its cellular compatibility to L-929. Moreover, PHB/Gel/MTZ nanofibrous scaffolds can effectively augment the development of fibroblasts and inhibit *E. coli* and *S. aureus*. The drug release profile demonstrated that MTZ release from Z_2_@7:3 nanofibrous scaffold started with initial burst release, followed by sustained release profile up to 100 h. More significantly, in vivo studies have shown that topically applied PHB/Gel/MTZ (Z_2_@7:3) nanofibrous scaffold plays an important role in wound healing of mice, specifically by augmenting the (TGF-β) transforming growth factor gene expression level in wounds of mice and promoting myo-fibroblast formation. It was also found that Z_2_@7:3 nanofibrous scaffold could down regulate wound inflammation, reduce the interleukin-6 (IL-6) gene expression level and accelerate wound healing. As a result, we believe that the Z_2_@7:3 nanofibrous scaffold may serve as a novel and useful scaffold for skin tissue regeneration.

## Figures and Tables

**Table 1:** Thermal data of nanofibrous scaffolds PHB, 8:2, 7:3, Z_1_@8:2, Z_1_@7:3, Z_2_@8:2 and Z_2_@7:3, DS = decomposition stages.

Thermal Characters	Polymer Matrices (PHB/GEL) without Drug			Drug Loaded Polymer Matrices (Z@PHB/GEL)			
	10:0	8:2	7:3	Z_1_@8:2	Z_1_@7:3	Z_2_@ 8:2	Z_2_@7:3
**T_d_** **T_onset_ (°C)**	235.83	246.11	247.91	243.36	231.36	223.75	209.27
**T_max_** **50% Weight Loss Temp. (°C)**	257.67	276.18	282.14	277.76	276.97	273.59	272.56
**1st DS** **Temp. range (°C)**	25.84–217.93	24.2–69.06	27.56–73.36	18.12–63.34	25.72–77.76	26.31–72.96	16.06–89.68
**1st DS Weight** **loss (%)**	0.384	1.647	2.41	1.548	2.354	1.342	2.868
**2nd DS** **Temp. range (°C)**	217.93–272.27	69.06–225.10	73.36–229.87	63.34–227.96	77.76–205.58	72.96–193.42	89.68–199.33
**2nd DS Weight loss (%)**	82.635	1.250	1.175	2.221	1.406	1.393	1.781
**3rd DS** **Temp. range (°C)**	273.03–375.96	225.10–289.89	230.63–294	227.96–290.94	205.58–288.62	193.42–285.96	199.33–283.4
**3rd DS Weight loss (%)**	10.104	78.6	69.012	75.496	68.819	77.752	66.46
**4th DS** **Temp. range (°C)**	375.96–799.94	289.89–433.28	294–445.16	290.94–432.77	288.62–452.03	285.96–426.3	283.40–473.99
**4th DS Weight loss (%)**	6.685	11.678	17.46	11.141	16.808	10.52	15.711
**5th DS** **Temp. range (°C)**	------	433.28–799.81	444.4–799.79	432.54–799.1	452.03–799.89	427.07–799.91	473.99–799.1
**5th DS Weight** **loss (%)**	------	5.633	7.201	8.297	6.72	6.409	11.117

**Table 2:** Mean tensile strength, mean elongation at break, (%) porosity, Water uptake (%) and contact angle of nanofibrous scaffolds PHB, 8:2, 7:3, Z_1_@8:2, Z_1_@7:3, Z_2_@8:2 and Z_2_@7:3.

Sample Name	Mean Tensile Strength (MPa)	Mean Elongation at Break (%)	(%) Porosity	Water Uptake (%)	Contact Angle (°)
PHB	6.5 ± 0.3	11.5 ± 0.4	80 ± 0.32	180.2 ± 0.3	102.8 ± 0.6
8:2	6.61 ± 0.05	15.6 ± 0.3	71.2 ± 1	320.1 ± 0.2	52.73 ± 1.1
7:3	8.5 ± 0.03	10.5 ± 0.2	56.2 ± 1.07	315.2 ± 0.1	44.79 ± 1.2
Z_1_@8:2	2.5 ± 0.08	2.4 ±1.2	60.1 ± 0.1	320 ± 0.1	129.34 ± 0.7
Z_1_@7:3	4.1 ± 0.05	7.2 ± 0.5	65.2 ± 0.3	330 ± 0.2	62.12 ± 08
Z_2_@8:2	2.7 ± 0.06	2.8 ± 1.1	68.1 ± 1	335 ± 0.1	48.88 ± 1.2
Z_2_@7:3	2.6 ± 0.03	3.9 ±1.4	72.4 ± 0.2	350 ± 0.2	35.2 ± 0.9

**Table 3:** Wound closure rates of nanofibrous scaffolds-treated groups and untreated positive control.

Wound Diameter (mm) (Mean ± SDM) and% of Wound Closure (W.C.)		Observation Day				
		Zero Day	Day 3	Day 7	Day 10	Day 14
**Positive** **Control (sterile gauze)**	Diameter	15.55 ± 0.39	14.1 ± 0.56	12.08 ± 0.28	8.56 ± 0.38	5.5 ± 0.44
	% W.C	0	17.39	39.41	69.53	87.39
**7:3**	Diameter	15.56 ± 0.25	13.86 ± 0.30	11.05 ± 0.13	9.15 ± 0.45	0.5 ± 0.58
	% W.C	0	22.24	49.82	65.54	99.79
**Z_2_@7:3**	Diameter	15.46 ± 0.19	13.58 ± 13.58	9.85 ± 0.78	6.025 ± 0.32	0.4 ± 0.55
	% W.C	0	24.18	60.56	85.28	99.83

## Data Availability

The data presented in this study are contained within the article and the Supplementary Materials.

## Supplementary Materials

The following supporting information can be downloaded at: www.mdpi.com/article/10.3390/polym14030454/s1, Figure S1: SEM image of PHB 7% (*w*/*v*) under 5000× magnification; Figure S2: SEM image of PHB/Gel (8:2 *w*/*w*) 7% (*w*/*v*) under 5000× magnification; Figure S3: SEM image of PHB/Gel (7:3 *w*/*w*) 7% (*w*/*v*) under 5000× magnification.
