# Supplementary material for: Metronidazole Topically Immobilized Electrospun Nanofibrous Scaffold: Novel Secondary Intention Wound Healing Accelerator
Source: Polymers (Basel). 2022 Jan 23;14(3):454. doi: 10.3390/polym14030454 (PMC8840736; doi:10.3390/polym14030454)
Supplement: Supplementary file 1 [file polymers-14-00454-s001.zip › polymers-1561581-supplementary.pdf]

polymers

MDPI

Supplementary Materials:

Metronidazole Topically Immobilized Electrospun Nanofibrous Scaffold: Novel Secondary Intention Wound Healing Accelerator

Ahmed A. El-Shanshory [1,*], Mona M. Agwa [2], Ahmed I. Abd-Elhamid [1], Hesham M.A. Soliman [1], Xiumei Mo [3] and El-Refaie Kenawy [4,*]

[1] Composites and Nanostructured Materials Research Department, Advanced Technology and New Materials Research Institute (ATNMRI), City of Scientific Research and Technological Applications (SRTA-City), New Borg Al-Arab, Alexandria 21934, Egypt; ahm_ch_ibr@yahoo.com (A.I.A.-E.); h.soliman@srtacity.sci.eg (H.M.A.S.)
[2] Department of Chemistry of Natural and Microbial Products, National Research Center, Dokki, Giza 12622, Egypt; magwa79@gmail.com
[3] Key Laboratory of Science and Technology of Eco-Textile, Ministry of Education, College of Chemistry, Chemical Engineering and Biotechnology, Donghua University, Shanghai 201620, China; xmm@dhu.edu.cn
[4] Polymer Research Group, Chemistry Department, Faculty of Science, Tanta University, Tanta 31527, Egypt
* Correspondence: shansho.medo@gmail.com (A.A.E.-S.); ekenawy@yahoo.com (E.-R.K.)

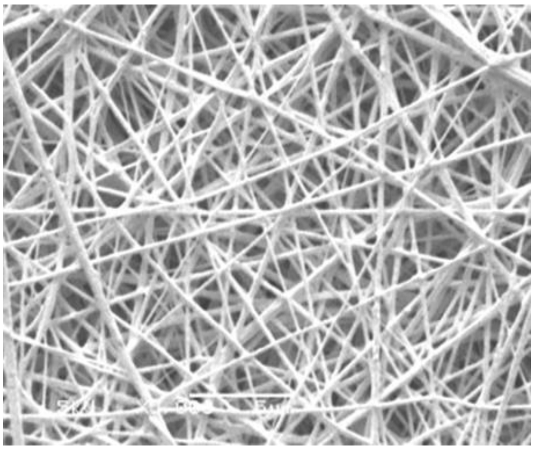

Figure S1. SEM image of PHB 7% (w/v) under 5000X magnification.

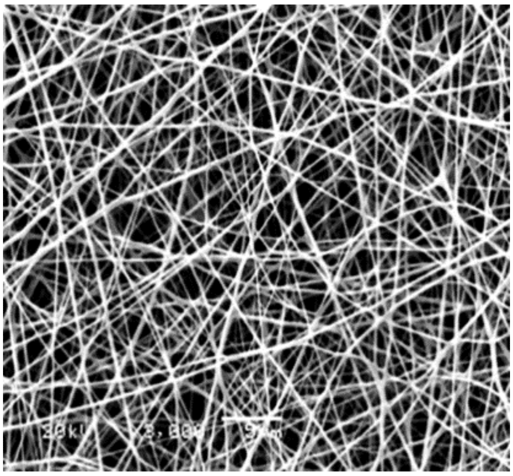

Figure S2. SEM image of PHB/Gel (8:2 w/w) 7% (w/v) under 5000X magnification.

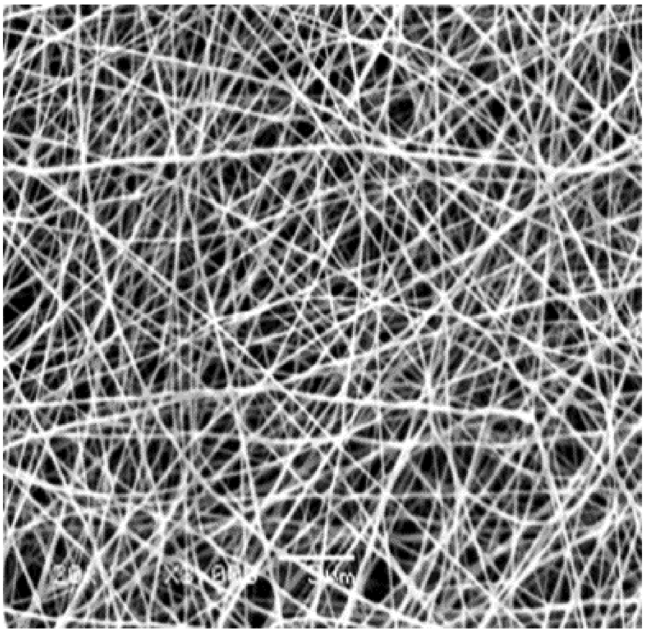

Figure S3. SEM image of PHB/Gel (7:3 w/w) 7% (w/v) under 5000X magnification.
